# Plasminogen-binding proteins as an evasion mechanism of the host’s innate immunity in infectious diseases

**DOI:** 10.1042/BSR20180705

**Published:** 2018-10-02

**Authors:** Dolores A. Ayón-Núñez, Gladis Fragoso, Raúl J. Bobes, Juan P. Laclette

**Affiliations:** Department of Immunology, Institute for Biomedical Research, Universidad Nacional Autónoma de México, Mexico City, Mexico

**Keywords:** enolase, fibrinolytic system, host/parasite relationship, immune evasion, Plasminogen

## Abstract

Pathogens have developed particular strategies to infect and invade their hosts. Amongst these strategies’ figures the modulation of several components of the innate immune system participating in early host defenses, such as the coagulation and complement cascades, as well as the fibrinolytic system. The components of the coagulation cascade and the fibrinolytic system have been proposed to be interfered during host invasion and tissue migration of bacteria, fungi, protozoa, and more recently, helminths. One of the components that has been proposed to facilitate pathogen migration is plasminogen (Plg), a protein found in the host’s plasma, which is activated into plasmin (Plm), a serine protease that degrades fibrin networks and promotes degradation of extracellular matrix (ECM), aiding maintenance of homeostasis. However, pathogens possess Plg-binding proteins that can activate it, therefore taking advantage of the fibrin degradation to facilitate establishment in their hosts. Emergence of Plg-binding proteins appears to have occurred in diverse infectious agents along evolutionary history of host–pathogen relationships. The goal of the present review is to list, summarize, and analyze different examples of Plg-binding proteins used by infectious agents to invade and establish in their hosts. Emphasis was placed on mechanisms used by helminth parasites, particularly taeniid cestodes, where enolase has been identified as a major Plg-binding and activating protein. A new picture is starting to arise about how this glycolytic enzyme could acquire an entirely new role as modulator of the innate immune system in the context of the host–parasite relationship.

## Introduction

Infectious agents migrate to their predilection sites in the host tissues, sometimes requiring to trespass physical barriers of the host such as epithelia, extracellular matrices (ECM), basement membranes, or circumvent several effector systems along their journey through the bloodstream [1–3]. They evade innate and adaptive host’s immune responses, involving the participation of multiple proteins, including proteolytic enzymes, receptors, immunomodulatory molecules, amongst many other factors that facilitate their dissemination and establishment in host’s tissues [4–8]. Infectious agents can uptake and use host proteins for their benefit [9–11]. In particular, it has been proposed that they can take advantage of the host’s coagulation cascade through the activation of plasminogen (Plg) to be converted into an active proteolytic enzyme (plasmin (Plm)). Plm participates indirectly in the degradation of ECM proteins and cell-junction proteins, thus facilitating invasion and establishment [12,13].

The goal of the present review is to list, summarize, and analyze different examples of Plg-binding proteins used by infectious agents to invade and establish in its host. These appear to be adaptive mechanisms of those infectious agents taking advantage of host’s proteins. To facilitate the analysis, the review was divided in bacterial and fungal infectious agents and protozoal and helminth parasites. A short section also considers tumor cells as invasive agents. Special emphasis was given to the mechanisms that helminth parasites, particularly cestodes, use to migrate and establish into predilection tissues in the host. Understanding these mechanisms might result in strategies for the prevention and control of infectious pathogens.

## Coagulation, complement, and fibrinolysis

### The coagulation cascade and the fibrinolytic system

**Figure 1:**
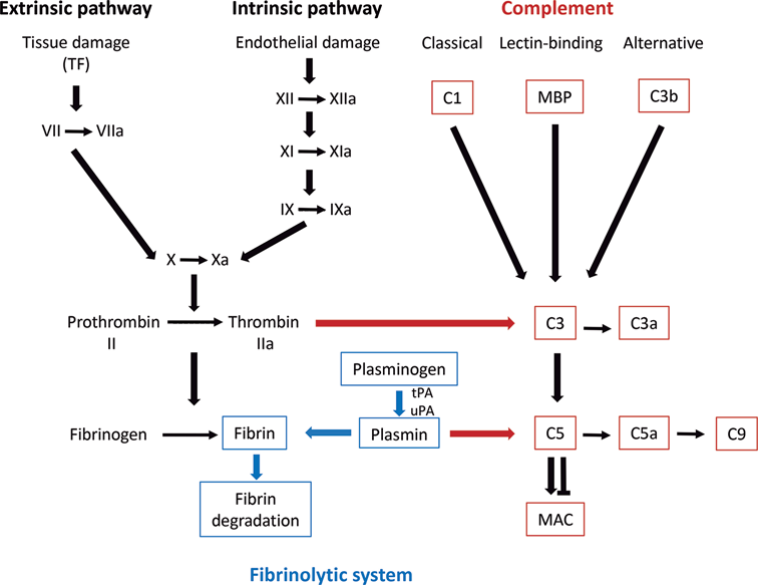
Relationship of coagulation and complement cascades with the fibrinolytic system The coagulation cascade has two pathways: the intrinsic and the extrinsic. Both pathways merge through factors V and X, resulting in the formation of clots. The fibrinolytic system relates with the final stage of the coagulation cascade, and its primary function is the proteolytic elimination of clots on blood vessels. Complement C5 can be activated by several coagulation enzymes (thrombin, factor IXa, factor Xa, factor XIa, and kallikrein). Plm can also activate complement through C5 degradation.

The coagulation cascade is a complex sequence of proteolytic reactions that ends with the formation of the fibrin clot. The coagulation cascade involving cellular (platelets) and proteolytic factors is activated when the endothelium of a blood vessel is damaged. The immediate goal is to stop bleeding, facilitating and promoting other mechanisms for damage control and repair. The coagulation cascade proceeds in two pathways: the intrinsic, formed by factors VIII, IX, XI, XII and the extrinsic, regulated by tissue thromboplastin and factor VII (Figure 1). Both pathways merge through factors V and X, that require calcium and platelet phospholipids, resulting in the formation of fibrin networks known as clots [14,15].

The fibrinolytic system participates in the final stage of the coagulation cascade and its primary function is the elimination of clots deposited in the blood vessels mainly through proteolytic action. The central reaction of the fibrinolytic system is the activation of Plg to Plm [16]. Degradation of clots depends on the binding of Plg/Plm to lysine residues located at the C-terminal end and to some internal lysine residues in fibrin networks (and other receptors); Plg binding requires lysine-binding sites (Figure 1) located in Kringle domains [17].

Plm is a broad-spectrum serine protease that degrades fibrin, ECM, and connective tissue through the participation of other proteolytic enzymes, including metalloproteases and collagenase [3]. A large number of pathogens including parasites express Plg receptors that immobilize Plg on their surface resulting in its activation; it has been proposed that the activation of Plg facilitates migration and invasion of these pathogens to different tissues in the host, as well as evasion mechanisms of the immune response, mostly through activation of the complement cascade [18–23].

### The coagulation cascade and the complement system

The coagulation and complement cascades are closely associated. A number of studies have demonstrated that coagulation and complement share several activators and inhibitors [24–28]. The complement cascade activation occurs by three distinct but interrelated pathways: the classical, the lectin, and the alternative (Figure 1). The classical pathway is initiated by immune complexes, the lectin pathway is initiated through the binding of the mannose-binding protein (MBP) to bacterial surfaces, and the alternative pathway is initiated by bacterial endotoxin present in the outer surface of bacteria and yeasts [29]. All pathways merge at the level of the C3 convertase before resulting in the formation of the membrane attack complex (C5b-9 or MAC) and the release of several active anaphylatoxins, opsonins, and other active molecules. The complement C5 can be activated by several coagulation enzymes including thrombin, factor IXa, factor Xa, factor XIa, and kallikrein (Figure 1). Plm can also activate complement but degrade C5, thereby preventing C5b deposition and MAC formation, which is a powerful lytic agent in bacterial infections. Both cascades contain a sequence of serine-proteases present in the plasma and serve important roles in innate host defense and hemostasis.

### Structure of Plg

**Figure 2:**
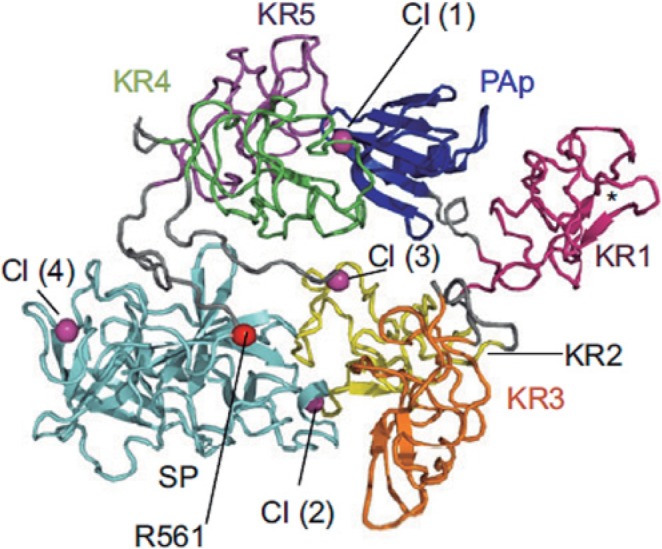
The structure of human Plg Domains are labeled and colored as follows: Pap, blue; KR1, pink; KR2, yellow; KR3, orange; KR4, green; KR5, purple; SP, cyan. The chloride ions (Cl) 1 and 2 are in the interface KR4/PAp and KR2/SP, respectively, and are shown as spheres. Two other chloride ions 3 and 4, bind to the KR2 and SP domain, respectively. The position of the activation loop is marked with a red sphere. The LR of KR1 is marked with an asterisk (*). Figure taken from Law et al. (2012) [38].

Plg is synthesized in the liver as a glycoprotein of 810 amino acids and approximately 90 kDa, also known as Glu-Plg. When secreted into plasma, the signal peptide in the N-terminal end (19 amino acid residues) is lost to become the mature form [30]. Plg can be found in two forms: the Glu-Plg that has a residue of glutamic acid at the N-terminal end and the Lys-Plg having a Lys^77^ residue at the N-terminal end [31]. Glu-Plg is converted into Lys-Plg by exogenous Plm that removes a 77 amino-terminal peptide [32]. Lys-Plg is more efficiently activated by fibrinolytic activators than Glu-Plg [33,34]. Both forms of Plg are made up of seven structural domains, an activation peptide in the N-terminal region known as the PAp domain (1–77 aa), five Kringle domains (KR1–5), and an SP serine protease domain (562–791 aa) (Figure 2) [35,36]. The Kringle domains mediate Plg-binding by lysine-binding sites (see above), to substrates and to cell surface receptors. The PAp domain interacts with KR4 and KR5, this interaction is critical to maintain a closed conformation of Plg. However, Plg can also be present in its open conformation (pre-activation), suggesting that a conformational rearrangement exposes the cleavage site for the Plg activators (PAs), whose action will result in the formation of Plm, the active protease [36,37].

### Activators and physiological inhibitors of Plg/Plm

The activation of Plg to Plm is mediated by the proteolytic action of two major types of PAs, the tissue-type (tPA) and the urokinase-type (uPA); both activate Plg by cutting specifically between Arg^560^–Val^561^ residues, in the SP domain [38]. However, some bacteria secrete different PAs, such as streptokinase (Streptococci, groups A, C, and G), staphylokinase (*Staphylococcus aureus* lysogenic), Pla (*Yersinia pestis*), PauA (secreted by *Streptococcus uberis*), and PadA (*Streptococcus dysgalactiae*) [39–44]. On the other hand, fibrinolysis is a highly regulated process involved in hemostasis, requiring participation of different inhibitors; best known are α2-antiplasmin and α2-macroglobulin (Plm inhibitors), PAI-1, PAI-2, and PAI-3 (inhibitors of Plg activators) [45–49].

### Participation of Plg/Plm in other cellular processes

In addition to its interaction with fibrin, Plg/Plm can act on other proteins such as cell surface receptors, coagulation components (factors V, VIII, and X), metalloproteases, as well as structural components of the ECM, including laminin, fibronectin, complement factors (C3 and C5), vitronectin etc. [13,50–54]. Therefore, Plg/Plm have been associated with several physiological and pathological functions in fibrinolysis and hemostasis, degradation of ECM, tumor growth, invasion, migration, tissue remodeling, wound healing, angiogenesis, and evasion of the immune response [13,55,56].

## Role of Plg receptors in sterile and non-sterile conditions

### Plg receptors in cancer

Cellular Plg/Plm receptors are ubiquitous, show high affinity for their ligand, and are usually expressed on cell surfaces [1,57,58]. Neoplastic cells behave in several aspects like infectious agents, indeed, Plg-binding proteins have also been involved as a mechanism to evade the innate response against tumor cells. Degradation of ECM is a crucial step in tumor cell invasion and thus, in metastasis. Plg is one of several proteases that facilitate tumor cell motility by disrupting the basement membrane and stromal barriers [59,60]. The presence of actin, enolase-1, cytokeratin 8, and annexin 2 have been associated with poor prognosis and resistance to chemotherapy of malignant tumors in patients. These proteins are overexpressed in cancer cells and have the ability to bind Plg/Plm, making them good diagnostic and prognostic markers, for example in breast, lung, and pancreas carcinomas [58,61]. The critical role of the Plg/Plm system in cancer biology is supported by *in vitro* and *in vivo* studies; α-enolase has been identified as a potentially useful candidate for diagnosis and prognosis as well as for therapy using antibodies [55]. *In vitro* treatment of lung and bone cancer cells with antibodies against α-enolase, as well as with shRNA plasmids, appears to be a promising approach to suppress tumor metastasis, as it inhibits ECM degradation and invasion of cancer cells [13,55]. Moreover, *in vivo* studies of cancer utilizing Plg-deficient mice, demonstrated a markedly reduced angiogenesis and decreased metastatic potential [62–64].

### Plg receptors in bacteria

Recruitment of host proteases on the bacterial surface represents a particularly effective mechanism for increasing invasiveness [65]. One of the protease systems involved is the Plg/Plm; for which over 40 binding proteins have been reported in bacterial species (Table 1) [57]. These proteins include metabolic enzymes, components of signaling pathways, structural proteins, amongst others. In *Mycobacterium tuberculosis* 13 proteins have been reported, 11 in *Borrelia burgdorferi*, and 13 in *Leptospira interrogans*, to mention a few examples. Several models have been proposed in bacterial infections to explain the involvement of these proteins during invasion [2,57]. The degradation of ECM proteins in different bacteria was also evaluated, for example: in *Leptospira*, bound Plg is converted into Plm by uPA, for degradation of fibronectin and laminin, as evaluated by ELISA [66]. Also, *Leptospira* enolase-bound Plg has been described to degrade vitronectin [12]. Several examples of Plg receptors have also been described for *Mycoplasma* species [67,68].

Plg/Plm regulates both, coagulation and complement cascades in bacterial infections; interaction with the complement system may help bacteria to evade host’s immune system, facilitating invasion. Plm cleaves human complement proteins C3b and C5 in the presence of *L. interrogans* proteins: LigA and LigB [69]. Moreover, Lsa23 can block activation of both, alternative and classical pathways of complement. PLG bound to Lsa23 could be converted into Plm, which in turn degrades C3b and C4b [70]. These results suggest that Lsa23 might be involved in complement evasion processes by acting on three different mechanisms and could assist *Leptospira* to overcome lysis promoted by the MAC.

**Table 1:** Plg-binding proteins in pathogenic bacteria

Plg-binding proteins	Bacterial species	References
Type 1 fimbriae	*Escherichia coli*	[71]
OspA	*Borrelia burgdorferi*	[72]
BBA70		[73]
OspC		[74]
CRASP-1, 3, 4, and 5		[75]
ErpP, ErpA, and ErpC		[76]
Erp63	*Borrelia spielmanii*	[77]
DnaK, GroES, GlnA1, Ag85 complex, Mpt51, Mpt64, PrcB, MetK, SahH, Lpd, Icl, Fba, and EF-Tu	*Mycobacterium tuberculosis*	[78,79]
LenA	*Leptospira interrogans*	[80]
Leptospiral surface adhesion, Lsa66 and Lp30		[81]
LIC12238, LIC10494, LIC12730, LipL32, LipL40, Lp29, Lp49, Lsa20 and Lsa6		[82]
EF-Tu		[83]
Lsa44 and Lsa45		[84]
GAPDH	Group A streptococci	[85]
	*Streptococcus pneumoniae*	[86,87]
	*Bacillus anthracis*	[88]
	*Lactobacillus crispatus*	[89]
	*Lactobacillus plantarum*	[90]
	*Clostridium perfringens*	[91]
	*Erysipelothrix rhusiopathiae*	[92]
	*Riemerella anatipestifer*	[93]
	*Escherichia coli*	[94]
Enolase	*Neisseria meningitidis*	[95]
	*Borrelia burgdorferi*	[96]
	*Mycoplasma gallisepticum*	[67]
	*Trichomonas vaginalis*	[97]
	*Candida albicans*	[98]
	*Lactobacillus crispatus*	[89]
	*Lactobacillus plantarum*	[99]
	*Leptospira interrogans*	[12]
	*Mycobacterium tuberculosis*	[100]
	*Mycoplasma pneumoniae*	[101]
Triosephosphate isomerase	*Staphylococcus aureus*	[102]
Phosphoglycerate kinase	Group B Stretococcus	[103]
Fructose 1,6-bisphosphate aldolase	*Mycobacterium tuberculosis*	[78,79]
	*Neisseria meningitidis*	[104]
DNaK and Peroxiredoxin	*Neisseria meningitidis*	[105]
PdhA-C, GAPDH-A, Ldh, Pgm, Pyk, and Tkt	*Mycoplasma pneumoniae*	[68]
Skizzle	*Streptococcus agalactiae*	[106]

Abbreviations: Antigen 85, mycolyltransferase, Fn binding protein, Ag85A, Ag85B, and Ag85C; DnaK, heat shock protein 70 or protein chaperone DnaK; EF-Tu, iron-regulated elongation factor TU; GAPDH, glyceraldehyde-3-phosphate dehydrogenase; GlnA1, glutamine synthetase A1; GroES, 10-kDa chaperonin; CRASP, surface protein that acquires the complement regulator; Icl, isocitrate lyase; Ldh, lactate dehydrogenase; LHP, dihydrolipoamide dehydrogenase; MetK, methionine adenosyltransferase; Mpt51, related Ag85 complex protein, with mycolil transferase, Fn binding protein D; Mpt64, immunogenic protein; OspA, outer surface protein A; OspC, outer surface protein C; PdhA-C, pyruvate dehydrogenases A to C; Pgm, phosphoglycerate mutase; PrcB, proteasome β subunit;Pyk, pyruvate kinase; Tkt, transketolase.

### Plg receptors in fungi

Several fungal pathogenic species express molecules that interact with host proteins during pathogen invasion, colonization, and growth. The ability to interact with host components, including blood, ECM proteins, and human complement regulators, appears to be essential for pathogen survival. Fungal parasite species express Plg-binding proteins (Table 2). *Candida* species have been reported to exhibit numerous Plg-binding proteins: eight proteins have been reported in *Candida albicans:* phosphoglycerate mutase, alcohol dehydrogenase, thioredoxin peroxidase, catalase, the transcription elongation factor, glyceraldehyde-3-phosphate dehydrogenase (GAPDH), phosphoglycerate kinase, and fructose bisphosphate aldolase [107]; four proteins have been reported in *C. parapsilosis:* CPAR2_404780, CPAR2_404800, Ssa2, and 6-phosphogluconate dehydrogenase 1 [108]. In the case of *Cryptococcus neoformans*, 18 proteins have been identified as capable of binding host’s Plg system to allow the fungus to cross tissue barriers, supporting the hypothesis that Plg binding may contribute to trespass the blood–brain barrier [109]. The role of enolase in pathogenicity has also been studied in fungal parasites including *Aspergillus nidulans, C. albicans* [110], *Paracoccidioides brasiliensis* [111,112], and *Pneumocystis carinii* [113]. However, only the role of enolase in the processes of invasion and dissemination of fungal infections has been hypothesized, since no functional studies have been done yet.

**Table 2:** Plg-binding proteins in pathogenic fungi

Plg-binding proteins	Fungi species	References
Pgm, alcohol dehydrogenase, thioredoxin peroxidase,catalase, transcription elongation factor, GAPDH, phosphoglycerate kinase, and fructose bisphosphate aldolase	*Candida albicans*	[107]
Pra1		[114]
Pgm		[115]
CPAR2_404780, CPAR2_404800, Ssa2, and 6-phosphogluconate dehydrogenase 1	*Candida parapsilosis*	[108]
Fructose 1,6-bisphosphate aldolase	*Paracoccidioides sp.*	[116]
Enolase	*Aspergillus nidulans* and *Candida albicans*	[110]
	*Paracoccidioides brasiliensis*	[111,112]
	*Pneumocystis carinii*	[113]
Thioredoxin-dependent peroxide reductade, heparinase	*Trichosporon asahii*	[117]
Triosephosphate isomerase	*Cryptococcus neoformans*	[118]
Not identified		[119]
Hsp70, Cpn60, glucose-6-phosphate isomerase, ATP synthase subunit β, Pyk, ATP synthase subunit α, response to stress-related protein, phosphoglycerate kinase, putative uncharacterized protein, ATP synthase γ chain, ATP synthase δ chain, Putative uncharacterized protein, ketol-acid reductoisomerase, Transaldolase, inorganic diphosphatase, dihydrolipoyl dehydrogenase, fructose-bisphosphate aldolase, glutamate dehydrogenase, enolase		[109]

Abbreviations: Cpn60, heat shock protein 60; Hsp70, heat shock protein 70; Pgm, phosphoglycerate mutase; Pyk, pyruvate kinase.

### Plg receptors in protozoan parasites

The role of Plg for intracellular parasites has been less documented (Table 3). However, involvement of Plg in invasiveness and pathogenesis of some parasites has been clearly shown. For example, it has been reported that binding of Plg/Plm contributes to virulence in *Leishmania mexicana*. Furthermore, Plg binding has been shown to be highly heterogeneous amongst different morpho-phenotypes of promastigotes, including a Plg-binding increase related to the differentiation of the promastigotes [120]. The course of the infection was also evaluated in a Plg-deficient mice, demonstrating that Plg has an effect on the distribution pattern of these parasites in the lesion produced by *L. mexicana*, but does not have an effect on the dissemination of the parasite to other organs [121]. *L. mexicana* enolase has been described to interact with Plg on the surface of the parasite through an internal motif: ^249^AYDAERKMY^257^ [122,123]. An activated C-kinase (LACK, *Leishmania* homolog of receptors for activated C-kinase) also binds Plg; this is a homologous receptor of *Leishmania sp.* that binds and activates Plg in the presence of tPA through an internal motif similar to that in enolase (^260^VYDLESKAV^268^); this being a new function of the protein that could contribute to the invasiveness of the parasite [124].

**Table 3:** Plg-binding proteins in protozoan parasites

Proteins	Parasite species	Binding characteristics	References
Enolase	*Leishmania mexicana*	- Heterogeneous binding between the morpho-phenotypes of promastigotes - Enolase binds through an internal motif (^249^AYDAERKMY^257^)	[120,122]
LACK		LACK binds through an internal motif similar to that of enolase (^260^VYDLESKAV^268^)	[124]
Enolase	*Plasmodium yoelii*	The oocysts bind the Plg	[125]
Enolase	*Plasmodium falciparum*	The enolase of the oocysts binds Plg through an internal motif (DKSLVK)	[126]
	*Plasmodium berghei*		
Not identified	*Trypanosoma cruzi*	The trypomastigote and epimastigote bind Plg on its surface	[127,128]
Not identified	*Trypanosoma evansi*	The Plg have greater bonding capacity compared with others from the same family	[129]
GAPDH	*Trichomonas vaginalis*	Natural GAPDH and the recombinant bound to immobilized Plg, FN, and collagen	[130]

On the other hand, *Trypanosoma cruzi* during its life cycle alternates between different morphological types: epimastigote, metacyclic trypomastigote in the insect vector, amastigote, and the blood trypomastigote in the mammalian host. The trypomastigote and the epimastigote thrive outside a host cell, which means that they interact directly with host fluids; both show the ability to interact with Plg [127]. This has also been demonstrated and quantitated in epimastigotes [129]. However, *T. evansi* possess receptors with higher Plg-binding affinity, unlike *T. cruzi* and other parasites of this family [121].

In the *Plasmodium* species, the oocysts play an important role in the host’s invasion. The oocysts have to trespass two physical barriers in the insect host: the peritrophic matrix and the midgut epithelium [126]. Enolase in *Plasmodium yoelii* is associated with nuclear elements, cell membrane, and cytoskeleton, suggesting that it may play non-glycolytic functions such as participating in the host invasion through Plg binding [125]. It was recently reported that the superficial enolase of the *P. berghei* and *P. falciparum* oocysts appear to facilitate attachment of the oocysts to the midgut epithelium in the insect, as well as of recruiting Plg through binding to an internal enolase motif (DKSLVK); this interaction is essential for the invasion of the parasite (activated Plg) and for the formation of oocysts [126]. In addition, other components of the fibrinolytic system have been involved in the infection of *P. falciparum*, such as uPA, which binds on the surface of malaria-infected erythrocytes and could be involved in the merozoite release process [131]. The uPA has also been involved in *Toxoplasma gondii* infection through a specific receptor (uPAR: uPA receptor), which could be implicated in macrophage rolling and infection through the expression and secretion of MMP-9 metallopeptidase complexes [132].

### Plg receptors in helminth parasites

The study of Plg-binding proteins in helminth parasites has been addressed in recent years (Table 4). Most of the studied parasite diseases have a life stage in the circulatory system, in contact with proteins of the fibrinolytic system of the host. Parasites have developed different strategies to evade the immune response of the host; one of them appears to be the recruitment of Plg on the worm’s surface. Plg-binding has been studied in *Dirofilaria immitis*; an E/S antigen extract of adult worms allowed identification of ten Plg-binding proteins: HSP60, actin-1/3, actin, actin 4, transglutaminase, GAPDH, Ov87, LOAG 14743, galectin, and P22U [7]. Moreover, an extract of surface proteins from adult worms of *D. immitis* identified eleven proteins, including only two of the abovementioned group: actin-5C, actin-1, enolase, fructose-bisphosphate aldolase, GAPDH, MSP protein domain, MSP 2, β-binding lectin-galactosidase, galectin, protein containing the immunoglobulin I-set domain, and cyclophilin Ovcyp-2. It has been suggested that they interact with the host’s fibrinolytic system during invasion [8]. GAPDH and galectin (rDiGAPDH and rDiGAL) recombinants of *D. immitis* were analyzed as Plg-binding proteins. Results indicated that rDiGAPDH and rDiGAL are able to bind Plg and stimulate the generation of Plm by tPA; this interaction requires participation of lysine residues. They also increased the expression of uPA in canine endothelial cells in culture, which suggests that they promote a favorable habitat free of clots in the intravascular environment of the parasite [133].

**Table 4:** Plg-binding proteins in helminth parasites

Proteins	Parasite species	Binding characteristics	References
Enolase	*Onchocerca volvulus*	Ov-ENO binds Plg	[134]
GAPDH		Ov-GAPDH	[135]
GAPDH	*Clonorchis sinensis*	rCsGAPDH and rCsANXB30 were able to interact with human Plg in a dose-dependent manner. The interaction could be inhibited by lysine	[136]
Annexin B30			[137]
Enolase	*Fasciola hepatica*	Present in the E/S products	[138]
HSP60, actin-1/3, actin, actin 4, transglutaminase, GAPDH, Ov87, LOAG 14743, Galectina and P22U	*Dirofilaria immitis*	In an extract of excretion/secretion antigens of adult worm of *D. immitis*	[7]
Actin-5C, actin-1, enolase, Fba, GAPDH, protein domain MSP, MSP 2, β-galactosidase binding lectin, Galectina, and cyclophilin Ovcyp-2		In a surface protein extract of adult worms	[8]
Enolase, Actin, GAPDH, ATP: guanidine kinase, Fba, Pgm, Triosephosphate isomerase, adenylate kinase	*Schistosoma bovis*	In a total extract of worm proteins	[139]
Enolase	*Echinostoma caproni*	Present in the E/S products	[20]
Enolase	*Taenia multiceps*	TmEno is a Plg receptor	[140]
Enolase	*Taenia pisiformis*	rTpEno could bind to Plg and could be converted into active Plm using host-derived activators. Its binding ability was inhibited by ɛACA	[22]
Enolase	*Taenia solium*	Plg-binding proteins of cysticerci; TsEnoA is a Plg receptor	[141,142]
Fascicilin-1, Fasciclin-2, MAPK, Annexin, Actin, and cMDH			[142]

Abbreviations: cMDH, cytosolic malate dehydrogenase; Fba, fructose-bisphosphate aldolase; MAPK, mitogen-activated protein kinase; Pgm, phosphoglycerate mutase; ɛACA, ɛ-aminocaproic acid.

On the other hand, in a total protein extract of *Schistosoma bovis* adult worms, ten Plg-binding proteins were identified: enolase, actin, GAPDH, ATP: guanidine kinase, fructose bisphosphate aldolase, phosphoglycerate mutase, triosephosphate isomerase, adenylate kinase and two hypothetical proteins of *S. japonicum* [139]. Recombinant annexin and enolase possess the ability to bind and activate Plg, suggesting that they play a role in the maintenance of hemostasis within the blood vessels [21,143]. In the case of cestodes, seven Plg-binding proteins were identified in *Taenia solium* cysticerci: fascicilin-1, fasciclin-2, enolase, mitogen-activated protein kinase (MAPK), annexin, actin, and cytosolic malate dehydrogenase [142]. Recombinant enolase was characterized and showed a strong Plg-binding and activating activity *in vitro*, suggesting that enolase could play a role in parasite invasion [141,142].

Other examples of helminth infections where parasite proteins have been involved in Plg/Plm binding as an evasion mechanism of the host’s innate defensive response are *Clonorchis sinensis*, in which GAPDH [136] and annexin B30 have been reported as Plg-binding proteins [137]. Enolases have also been reported as Plg-binding proteins in *Onchocerca volvulus* [134], *Fasciola hepatica* [138], *Taenia multiceps* [140], and *T. pisiformis* [22].

## Plg–enolase interaction

Enolase is perhaps the most studied Plg-binding protein in different organisms. Enolase has been identified as an octamer on the surface of group A streptococci; molecular docking analysis have revealed the fine detail of the Plg-enolase binding. Interaction with KR1 and KR5 domains of Plg occurs through lysine residues located at the C-terminal end of enolase, as well as on another internal binding site. Plg undergoes a conformational change to expose the cut site for PAs in order to induce Plm formation [144].

**Figure 3:**
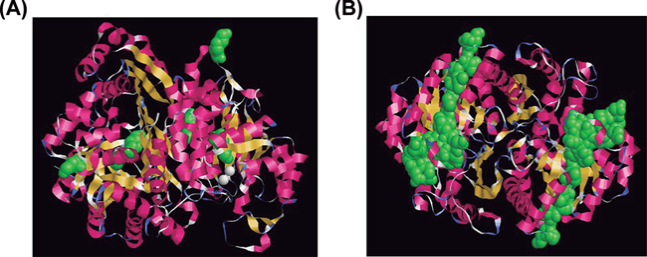
Molecular modeling of *T. solium* enolase A (TsEnoA) showing Plg binding sites (**A**) Identification of the C-terminal lysine residues are shown in green; (**B**) identification of the internal Plg-binding motif of *T. solium* enolase (also shown in green). The modeling was done in: http://www.openrasmol.org/.

Molecular docking studies have not been carried out for Plg–enolase in parasites; putative Plg-binding sites have been proposed by their similarity with described bacterial binding sites. *T. solium* enolase (TsEnoA) has been shown to bind Plg [142]; apparently, the internal site of Plg is involved but not the lysine residues at the C-terminal end. This idea is supported by results of assays using ɛ-aminocaproic acid (ɛACA), a synthetic inhibitor of the Plm–Plg system which binds to accessible lysine residues. In order to find out the spatial distribution of Plg-binding sites on TsEnoA, we used the amino acid sequence to predict the protein structure using Swiss-Model and RasMol programs. The lysine residues at the C-terminal end were not exposed (Figure 3A), in contrast with the internal site that appears entirely accessible (Figure 3B).

## Modulation of Plg/Plm function by enolase as a mechanism against host’s innate responses in taeniid parasites

Parasites have developed an intimate molecular relationship with their hosts through evolution, thus requiring a number of host proteins for survival, for example to complement their metabolism [10,133]. It is also well known that although parasites have a vast repertoire of proteases [145,146], they also appear to take advantage of host’s proteases. We have localized TsEnoA binding of Plg/Plm on the surface of *T. solium* cysticerci [141,142]. As Plm has been involved in the degradation of fibrin clots and ECM, we proposed that binding and activation of Plg might help the parasite to colonize host tissues. Recruitment and activation of Plg has been proposed as a mechanism involved in survival or establishment for other helminths [7,8,139].

Human and porcine cysticercosis is acquired by ingestion of *T. solium* eggs. Poor hygiene conditions and domestic management of human feces and especially, cohabitation with an adult-worm carrier are factors that facilitate transmission of the disease. Eggs contain hexacanth embryos surrounded by an impermeable and highly resistant envelope called embryophore, which allows survival under adverse environmental conditions. Once in the host’s gut, proteolytic enzymes and bile salts trigger the release and activation of the hexacanth embryo (also known as oncosphere). Activated embryos trespass the host intestinal wall and reach lymphatic and blood capillaries, through which they are distributed to a wide variety of predilection organs and tissues (subcutaneous tissue, skeletal and cardiac muscle, brain, eyes etc.) [147]. Although events occurring after embryos trespassing the intestinal wall remain mostly unexplored, it is known that few weeks are required for an oncosphere to transform into a metacestode known as cysticercus. The mechanisms by which the parasite reaches a predilection tissue, like the central nervous system, where cysticerci causes neurocysticercosis, are also unknown.

Taeniids possess adhesion molecules and metalloproteases able to degrade ECM [148]. Two previous reports have shown that enolase from *T. multiceps* and *T. psiformis* bind and activate Plg [22,140]. Enolase of *T. solium* was also found to be Plg-binding and activating protein [141,142]. Therefore, it appears that binding and activation of Plg might help early larval forms colonize host tissues, as Plm could aid degradation of fibrin clots and ECM. An interesting experiment would be to test treatment of *T. crassiceps* cysticerci embedded in Matrigel, using antibodies against α-enolase or shRNA plasmids to find out if cysticerci degrade ECM, following a similar strategy to that currently being tested against cancer treatment [13,55]. Thus, the role of parasite proteins that can bind and activate Plg, along with the extensive expression of proteases such as a chymotrypsin-like peptidase, trypsin-like and cathepsin B-like peptidases [149], could be more related to the capacity of parasites to enter through the intestinal mucosa and invade host tissues. We can speculate that Plm can exert an initial role during parasite invasion to host tissues; once established, it is possible that Plm and other proteases could participate in ECM degradation, allowing parasite establishment, growth, and development, as it has been reported for bacteria, protozoan, and helminth parasites. Moreover, no proteases capable of degrading fibrin clots have been found in parasites. *T. solium* being the only taeniid reaching the CNS, shows the expression of adhesion molecules specific for brain ligands that might be the main factors involved in this tissue-specific parasite invasion.

*T. solium* and other taeniids possess at least four enolase genes [142]. Except for TsEno4, tapeworm enolase amino acid sequences are not orthologs of vertebrate isoforms; thus, the origin of enolase isoforms in vertebrates and invertebrates is not monophyletic. TsEnoA has been characterized and expressed in bacteria showing a strong Plg-binding and activating activity *in vitro*. TsEno4 is considerably smaller: 28 compared with 46–49 kDa of the other three *T. solium* enolases. Preliminary results have shown that TsEno4 lacks enolase activity as well as Plg-binding activity (Ayón-Núñez et al., unpublished). As TsEno4 is the ancestral enolase in cestodes, the fact that it lacks enolase activity suggests that other isoforms fulfilled the need for a glycolytic enzyme function; TsEno4 lost its enzyme activity and perhaps is now involved in other moonlighting functions that are relevant for the parasite. Our current efforts are directed to explore this possibility. Regardless of the TsEno4 case, as Plg has also been implicated as a modulator of fibrinolysis, complement or even the immune response involved in the survival of a number of pathogens, a tantalizing question would be if this is also an adaptive mechanisms in taeniid parasites.

## Conclusion

Plg/Plm seems to play a relevant role in several examples of infectious agent relationships, including bacteria as well as protozoan, helminth, and perhaps taeniid parasites; possibly involved in the invasion and migration of the parasites through the tissues of the host. Understanding the interactions of different Plg-binding proteins in parasites will allow realizing a new mechanism of invasion, migration, and/or establishment that has not been addressed.

## Abbreviations

ECM: extracellular matrix
GAPDH: glyceraldehyde-3-phosphate dehydrogenase
MAC: membrane attack complex
PA: plasminogen activator
Plg: plasminogen
Plm: plasmin
tPA: tissue-type PA
uPA: urokinase-type PA
